# APOE status and its association to learning and memory performance in middle aged and older Norwegians seeking assessment for memory deficits

**DOI:** 10.1186/1744-9081-3-57

**Published:** 2007-10-31

**Authors:** Eike Wehling, Astri J Lundervold, Brit Standnes, Leif Gjerstad, Ivar Reinvang

**Affiliations:** 1 Department of Biological and Medical Psychology, University of Bergen, Norway; 2 Center for the study of human cognition, Department of Psychology, University of Oslo, Norway; 3 Department of Neurology, Buskerud Hospital, Drammen, Norway; 4 Department of Neurology, Rikshospitalet University Hospital, Oslo, Norway

## Abstract

**Background:**

We examined the hypothesis that deficits in learning, memory, and other cognitive functions are associated with the ε4 allele of the Apolipoprotein E (APOE) gene in a non-demented sample with memory complaints recruited from a population with a high prevalence of this allele.

**Methods:**

The study group comprised 70 consecutively referred patients aged 50–75 seeking assessment due to memory complaints. They were screened for dementia, for neurological and psychiatric disease, and for cerebral infarction using Magnet Resonance Imaging (MRI). Participants were classified as non-demented based on clinical evaluation and results on cognitive tests.

**Results:**

APOE ε4 carriers (56% of the sample) showed poorer performance than non-carriers on the Mini Mental State Examination, a number of measures of verbal memory function from the California Verbal Learning Test, and visual recall. In 46% of the participants, psychometric criteria for amnestic Mild Cognitive Impairment (aMCI) were satisfied.

**Conclusion:**

Findings may be partly explained by a significant number of participants being in a preclinical phase of Alzheimer's disease. The observed deficits in learning performance and the lack of significant age modulation of the genetic association suggest a more general genetic effect. The findings are consistent with known neurobiological function of APOE ε4, including both increased risk of neurodegenerative disease and reduced synaptic integrity in older age.

## Background

Alzheimer's disease (AD) is the most prevalent form of dementia in all age groups, with less than 1% incidence before age 65, but with an exponential increase with age [1]. Prior to diagnosis of AD many patients go through a clinical phase termed mild cognitive impairment (MCI), which has been characterized by subjective memory complaints and clinical criteria of cognitive impairment without being demented [2,3]. Memory may be the only cognitive function affected (amnestic MCI), but MCI may also affect other cognitive domains in isolation or in combination with memory impairment [3]. Patients with MCI are shown to be at high risk of progression to AD [4]. However, non-demented elderly persons with subjective memory complaints are found quite frequently in the population at large and are a heterogeneous group. Koivisto and collaborators [5] found a prevalence of 76% in a randomly selected population sample aged 60–78 years, with the highest frequency in the younger part of the sample. Studies of population prevalence of MCI in similar age groups, using age adjusted psychometric criteria of memory impairment, yield lower estimates, typically 2–5% [6,7].

The risk of developing AD is significantly increased by carrying one or more ε4 alleles of the Apolipoprotein E (APOE) gene [8], and the risk increases in a dose dependent manner in relation to debut of AD before age 70 [9,10]. APOE ε4 is also associated with reduced memory function in clinically defined MCI patients [11]. Smith and collaborators [12] studied a group of MCI patients diagnosed on the basis of memory deficits and found that APOE ε4 was associated with poorer performance on tests of learning and recall in MCI patients, but not in normal controls. They suggested that APOE-related memory deficit is a specific cognitive phenotype in patients with AD pathology. In a group of non-demented older adults, Bondi and collaborators [13,14] found memory impairment at study entry in APOE ε4 carriers, affecting measures of recall, recognition discriminability, and learning slope as measured by the California Verbal Learning Test (CVLT). No group differences between APOE ε4 carriers and non-carriers were found in other cognitive domains. Follow-up data [14] showed that a subgroup developed dementia, and that the risk was related to APOE status and memory performance at study entry.

APOE is a cholesterol transporting protein coded on chromosome 19, with complex functions [15] that may affect cognition and brain function in clinical as well as non-clinical groups. A meta-analysis of population based studies by Small et al. [16] concluded that the ε4 allele is associated with reduction in global cognitive functioning, episodic memory, and executive functioning. Their results revealed that APOE ε4 effects may vary with age, having a larger impact in middle aged individuals than in the very old. Longitudinal studies of elderly, healthy participants have found that APOE ε4 was associated with more rapid memory decline, but not with memory performance at any given time of testing [17,18]. Mortensen and Høgh [19] showed that the APOE ε4 allele was significantly associated with a decline in tests of speeded attention and visuo-construction in women, in particular those between 70 and 80 years of age. Evidence for an effect of APOE on brain function in healthy individuals is reinforced by studies using metabolic and functional brain imaging techniques [20-22] and reaction time based attention tasks [23,24].

The studies above indicate that the APOE ε4 allele may affect cognition and brain function in both clinical and non-clinical groups. Memory complaints are prevalent in the middle aged population, and the risk for developing AD based on memory complaints alone is low. The proportion of study participants with ε4 allele will also vary with recruitment and inclusion criteria in relation to base rates in the general population. Although APOE ε4 has been shown to be a risk factor for AD in many populations, the APOE ε4 - AD association was shown to be weaker among African Americans and Hispanics than in Caucasians and Japanese [10,25]. The prevalence of ε4 alleles also varies significantly between European populations, from a low incidence (10–15%) of ε4 alleles in southern European populations to a high incidence (40–50%) in northern European populations [26]. The functional consequences of the genetic variation have not been fully explored. One consequence might be a difference in incidence of early debut of AD between southern and northern European countries. This has not been confirmed by comparative epidemiological studies (ERODEM) [27], but methodological differences across the studies preclude definite conclusions.

In the present study we examined neuropsychological performance associated with variants of APOE alleles in non-demented middle aged and older Norwegian participants, who are part of a population with high incidence of APOE ε4. Earlier studies have reported a high prevalence of subjective memory complaints in 60–70 year olds [5] and a low risk of developing AD before age 75 [28]. This indicates that a clinically recruited non-demented group of participants younger than 75 years will include a significant proportion of participants with age related reduction of cognitive functions that are not primarily due to degenerative pathology. Based on the high prevalence of ε4 alleles in the Norwegian population [26], we expected a high incidence of these alleles in our study group, and we asked if the pattern of cognitive function confirmed the results in samples from populations with lower base rates of ε4.

## Methods

### Patients

Participants were referred by their primary physician to a clinical research project on age related cognitive impairment and risk for dementia at the Department of Neurology, Buskerud Hospital, Drammen, Norway. Only patients in the age range 50–75 years were candidates for the project. Each individual underwent an extensive assessment based on the Consortium to Establish a Registry on Alzheimer's Disease (CERAD) protocol [29], including a comprehensive neuropsychological investigation that was supplemented by the Norwegian version of the CVLT [30]. All patients underwent full neurological and medical examination, including Magnet Resonance Imaging (MRI) brain scan and laboratory tests. Inclusion criteria were complaints of memory problems of at least 6 months duration, an estimated IQ score >80, and a score of 24 or higher on the Mini Mental State Examination (MMSE) [31]. Patients with a neurological, psychiatric, or another diagnosis which might affect cerebral function were excluded as well as patients with a known history of alcohol or substance abuse. All cases were reviewed by a panel including a neurologist and a neuropsychologist after completion of the study protocol, and patients who received a dementia diagnosis according either to the DSM-IV [32] or the ICD-10 [33] criteria were excluded. The final sample included in the present study consisted of 70 individuals, 33 men and 37 women. The average age was 63.9 years (SD = 7.7). All participants had completed obligatory basic education (7 years in this cohort). The average number of years of education for the sample was 11.2 years (SD = 3.0), the average IQ was 106 (SD = 14), and the sample's average MMSE score was 27.89 (SD = 1.78). All individuals were living independently at the time of participation in this study. The study was performed according to the Declaration of Helsinki [34] on guidelines for biomedical research involving human subjects.

### Apolipoprotein E

The APOE genotype was determined with polymerase chain reaction (PCR) according to standard methods. Individuals were classified as APOE ε4-positive (ε4 carriers) or APOE ε4-negative (non-carriers) based on the presence or absence of at least one ε4 allele. The ε4 carrier group (56% of the study group) comprised 39 patients, with allele combinations ε 2/4 (n = 4), ε 3/4 (n = 24) and ε 4/4 (n = 11). Thus, 16% of the sample was ε 4/4 homozygote. In the non-carrier group of 31 patients the allele combinations were ε 2/3 (n = 4) or ε 3/3 (n = 27). No patient had the allele combination ε 2/2.

### Neuropsychological tests

The neuropsychological test battery was administered according to the CERAD protocol [29]. Main areas of cognitive functioning were assessed. The areas and test measures are shortly described below. For a more detailed description, see [35]. Since impaired performance on verbal memory acquisition and recall have been demonstrated particularly sensitive measures to discriminate between ε4 carriers and non-carriers and in addition are well known markers for identifying early stages of AD [13,14], we considered specific measures on tests of verbal memory function as primary outcome measures (i.e. total learning score, short and long delay free recall (all from the CVLT), Verbal Paired Associate Test – learning and recall). All other scores were regarded as background variables.

### Intellectual function

Two subtests (Vocabulary and Matrix Reasoning) from the Norwegian version of the Wechsler Abbreviated Scale of Intelligence (WASI) [36] were administered to estimate an IQ score. In the Vocabulary subtest, participants were asked to define orally presented words. In the Matrix Reasoning subtest, incomplete patterns were presented and the participants were asked to complete the patterns by pointing to one of five available response alternatives.

### Memory function

The California Verbal Learning Test (CVLT) [37] was included to obtain measures of verbal learning, recall and recognition, as well as learning strategies, error types, and serial position effects. In the learning task, the participants were presented to a list of 16 words (list A), where each word belonged to one of four categories. The list was presented and recalled five times before a second list (list B) was presented. The participants were immediately after the recall of list B asked to recall the words from list A, both in a free and cued recall condition. After an interval of 20 minutes, the participants were again asked to recall the words from list A in a free and cued recall and recognition condition. The Verbal Paired Associates Test [38] was included as another measure of verbal learning and memory function. 15 word pairs were presented, and the number of recalled pairs was recorded.

The Rey Complex Figure Test (RCFT) [39], recall condition, was included as a test of visual memory function. The participants were asked to copy the Rey-Osterrieth figure (a visuo-constructive task). After an interval of 20 minutes, the participants were then asked to draw the figure as they remembered it from the copy-task.

### Attention and psychomotor speed

Two visuo-motor tests of attention and psychomotor speed were included, the Digit Symbol Test [40] and the Trail Making Test A [41]. In the Digit Symbol Test, a sheet containing rows of blank squares were presented, each square being paired with a randomly assigned number (1–9). The task was to fill in as many blank squares as possible within 90 seconds. In the Trail Making Test A, the participants were requested to draw lines to connect consecutively numbered circles (1–25) as fast as possible. The time to complete the tasks was recorded.

The Trail Making Test B [41] was included as a measure of cognitive flexibility. The participants were asked to draw lines to connect circles by alternating between circles with numbers and letters (1-A-2-B etc.). The time to complete the test was recorded. The third subtest of a Stroop Color Word Test [42] was used as a measure of cognitive flexibility/inhibition. In the first subtest, the participants were asked to name color patches (1), then to read color words (2), and in the third condition to name the color of color-words printed in an ink of a different color (3) as fast as possible. Time to complete the tasks was recorded.

### Verbal function

Fluency of speech was assessed using the Controlled Word Association Test (COWAT)[43]. Participants were asked to name as many words as possible with a given first letter within one minute, using the letters F, A, and S. Afterwards, participants were asked to name as many animals as possible within a minute. The Boston Naming Test was used to provide information about ease and accuracy of word retrieval. 15 items of the Boston Naming Test [44] were presented, including five words with low, medium and high frequency of occurrence, respectively.

### Subjective memory complaints

A standardized interview was performed by the examining physician, according to a Norwegian translation of the CERAD Clinical History protocol. The information obtained from the patient was scored by the physician at the time of the interview. In addition to the two items on memory complaints and their impact on everyday life, the protocol itemized 7 specific domains of non-memory complaints.

### Amnestic MCI

Amnestic MCI (aMCI) was defined when a participant obtained a result on the long delay free recall CVLT subtest that was at least 1.5 standard deviations (SD) below the age and gender adjusted norm. These norms are the standard norms presented by the test-developers [37].

### MRI protocol

Magnet Resonance Imaging (MRI) was included to detect and exclude patients with gross morphological changes. Imaging was initially performed with a 0.5 T Philips scanner, replaced at a later stage with a Philips 1.0 T scanner. Standard T1 and T2 clinical scanning sequences were used and visually rated from a hard copy by the departmental chief radiologist, who was blinded with regard to other study parameters [45].

### Data analyses

For all statistical analyses, SPSS version 14.0 was used [46]. Independent samples t-tests were used to test the differences in demographic characteristics of the ε4 carrier and non-carrier groups. Categorical data was analyzed using chi-square test. When data met the assumption of normality, independent samples *t*-tests were used to examine group differences on neuropsychological measures. When basic assumption for parametric tests, i.e. normal distribution, was violated, Mann-Whitney *U *nonparametric tests were used. These analyses were completed with calculations of effect sizes, i.e. Cohen's *d *for parametric tests and *r *as an approximate effect size when nonparametric tests were utilized. Additionally, repeated measures ANOVAs were used to assess general effects of learning, retention and recall, respectively. Learning was assessed by entering the performance on learning trial 1 to 5 as within subject variables. Retention was examined by entering learning trial 5 and the delayed free recall score, and the recall measure included the scores on the short delay and long delay free recall subtests as within subject variables. All analyses tested interaction effects of genotype with gender and of genotype with age. The sample was split by the median (65 years) into two age groups, entering age and genotype as fixed factors. In case of a significant group effect between ε4 carriers and non-carriers, the dose effect was explored by comparing the results in ε4 homozygotes and ε4 heterozygotes. All statistical tests were two tailed. The alpha level was generally set at 0.05. Since several measures of cognitive functioning were included, it seemed appropriate to control for Type I error rate for the primary outcome measures, i.e. the measures of verbal memory function. For those analyses, correcting according to Bonferoni, a *p *value of 0.01 was regarded as statistically significant.

## Results

The demographic characteristics of the ε4 carriers and non-carriers are shown in Table 1. There were no significant group differences in age, education, or gender distribution between the group of ε4 carriers and non-carriers. MRI findings were noted in 67% of non-carriers and 71% of ε4 carriers. The frequency of MRI findings was not significantly different between the groups. The most frequent finding was subcortical hyperintensities, reported in 57% of the non-carriers and 47% of the ε4 carriers. Cortical or subcortical atrophy was found in 20% of the non-carriers and 27% of the ε4 carriers.

**Table 1:** Demographic characteristics in APOE ε4 carriers and non-carriers

	non-carriers (n = 31)	ε4-carriers carriers (n = 39)	*t*-values	*p*-values
	M (SD)	M (SD)		
Age at test	62.9 (8.81)	64.7 (7.15)	-0.979	0.331
Years of education	11.4 (3.11)	10.8 (2.98)	1.123	0.266
Sex (M/F)	16/15	17/22		0.631*

* Chi-square test statistic

Subjective memory problems of at least 6 months duration were present in all patients, and more than half of the sample (57%) reported duration of memory problems longer than 2 years. Typical mode of onset was gradual progression (59%), but 13% reported that the impairment had been stable, and the rest reported a fluctuating course. ε4 carriers did not complain of more severe memory problems or of having more non-memory cognitive problems than the non-carriers.

There were no statistically significant differences between the group of ε4 carriers and non-carriers on tests of intellectual function, verbal function, attention/psychomotor speed and visuo-constructive function (Table 2). There was, however, a significant difference in the MMSE score between the APOE groups, showing that ε4 carriers obtained a statistically significant lower performance score than the non-carriers (*p *= 0.032). Follow-up calculation of Cohen's *d *revealed an effect size of (*d *= 0.53), representing a medium sized effect according to Cohen's definition [47]. Statistically significant differences between the two APOE groups were also found on Boston Naming Test (*p *= 0.03, *r *= -0.26) and the RCTF-recall measure (*p *= 0.02, *r *= -0.27).

**Table 2:** Mean performance (SDs shown in parantheses) of APOE ε4 carriers and non-carriers on cognitive measures (raw scores)

	ε 4 non-carriers (n = 31)	ε 4 carriers (n = 39)	*t*-values/*U*-values	*p*-values	Effect sizes^a^
MMSE	28.4 (1.6)	27.5 (1.8)	2.19^c^	0.03	0.53
WASI-IQ	105 (13.4)^b^	106 (15.3)^b^	0.068^c,e^	0.95	0.01
Stroop Color Word Test (seconds)	94 (54.3)	106 (58.0)	-0.840^c^	0.40	0.22
WAIS-R Digit Symbol (items)	35 (10.5)	32 (12.1)	1.166^c^	0.25	0.27
FAS (items)	30 (12.0)	31 (12.8)	-0.262^c^	0.79	0.08
Animal fluency (items)	16 (5.8)	17 (5.6)	-0.299^c^	0.77	0.18
Trail Making A (seconds)	48 (22.0)	58 (26.9)	477^d^	0.13	-0.18
Trail Making B (seconds)	144 (112.6)	165 (89.4)	479.5^d^	0.14	-0.18
Boston Naming (items)	15 (1.1)	14 (1.1)	539.5^d^	0.03	-0.26
Rey Complex Figure Test – copy (points)	33 (5.4)	31 (7.0)	487^d^	0.16	-0.17
Rey Complex Figure Test – recall (points)	16 (9.0)	11 (7.5)	410.5^d^	0.02	-0.27

Degrees of freedom for all parametric tests was 68

^a ^For *t*-tests, Cohen's *d*, for nonparametric tests *r *were calculated

^b ^Standardized scores

^c ^Test statistics according to independent *t*-tests

^d ^Test statistics according to Mann-Whitney *U *tests

^e ^Test statistic based on age-adjusted scores

**Figure 1:**
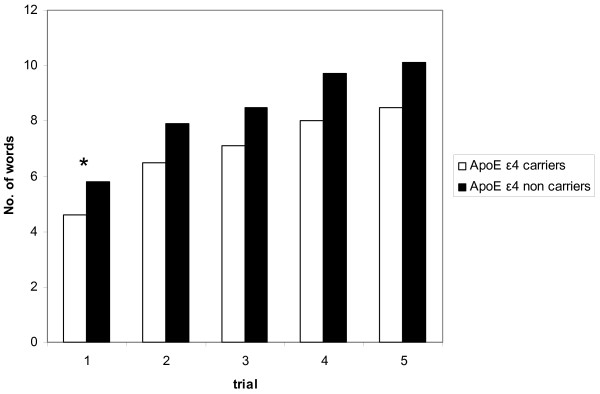
Mean number of words on the five learning trials from the CVLT in APOE ε4 carriers and non-carriers. * indicates *p *= 0.04.

ε4 carriers showed lower performance than non-carriers on several CVLT measures, as shown in Figure 1 and Table 3. Group differences became statistically significant only for Learning trial 1 but for none of the other comparisons; however, effect sizes revealed small to medium effects for several measures.

**Table 3:** Mean learning and memory performance (raw scores) on the Verbal Paired Associate Test and the California Verbal Learning Test (CVLT) in APOE ε4 carriers and non-carriers (SDs shown in parantheses)

	ε 4 non-carriers	ε 4 carriers	*t*-values/* U*-values	*p-values*	Effect sizes^a^
	(n = 31)	(n = 39)			
*Verbal Paired Associate Test – learning (errors)	33 (22.7)	41 (22.2)	-1.51^c^	0.14	0.52
*Verbal Paired Associate Test – recall (errors)	7 (4.2)	9 (4.8)	-1.80^c^	0.08	0.63
**CVLT**					
List A Trial 1 recall	5.8 (2.6)	4.6 (2.2)	2.14^b^	0.04	0.51
List A Trial 5 recall	10.1 (3.8)	8.5 (3.3)	1.85^b^	0.07	0.46
*Total learning (trial 1–trial 5)	42 (15)	34.7 (11)	2.34^b^	0.02	0.57
*Short delay free recall	7.3 (4.3)	6.1 (3.9)	1.20^b^	0.24	0.30
Short delay cued recall	9.3 (3.8)	8.1 (3.4)	1.35^b^	0.18	0.34
*Long delay free recall	8.5 (4.3)	6.8 (3.9)	1.78^b^	0.08	0.44
Long delay cued recall	9.1 (3.5)	8.1 (3.5)	1.18^b^	0.24	0.29
Percent recognition discriminability	89 (10.1)	84 (11.4)	1.84^b^	0.07	0.47
Intrusions learning	0.3 (0.7)	0.8 (1.4)	467^c^	0.03	-0.25
Short delay intrusions	0.2 (0.5)	0.3 (0.6)	559.7^c^	0.45	-0.09
Long delay intrusions	0.4 (0.6)	0.7 (1.5)	545^c^	0.39	-0.10
Recognitions hits	14.3 (1.8)	13.9 (1.9)	521.5^c^	0.32	-0.12
Recognition false alarms	3.0 (4.3)	4.7 (4.2)	413.5^c^	0.02	-0.27
Response bias (recognition)	0.09 (0.4)	0.24 (0.4)	462^c^	0.09	-0.20
Percentage consistency	75.7 (17.1)	67.6 (15.9)	395^c^	0.01	-0.30

Degrees of freedom for all parametric tests was 68

* According to Bonferoni correction, a *p*-value of 0.01 was regarded as significant for these scores

^a ^For *t*-tests, Cohen's *d*, for nonparametric tests *r *were calculated

^b ^Test statistics according to independent *t*-tests

^c ^Test statistics according to Mann-Whitney *U *tests

### Learning measures

A repeated measures ANOVA, including the five CVLT learning trials, showed that the non-carriers showed an overall better learning performance than ε4 carriers (F (1,68) = 5.46, *p *= 0.022). There was no interaction between group and learning trial. On average, participants in both APOE groups recalled more items on trial 5 than on trial 1. Consistent with this finding, the APOE groups showed no significant difference in slope of the learning curve, characterizing performance increment across trials (Figure 1). To explore strategies used in the learning process, we compared semantic and serial clustering, recall consistency, and serial position effects in the two APOE groups (Table 3). ε4 carriers showed a significantly lower consistency across learning trials (MW, *p *= 0.01, *r *= -0.3) as well as significantly more intrusions in the learning trials (MW, *p *= 0.03, *r *= -0.25). There was no significant difference between the groups regarding clustering strategies and serial position effects.

### Recall and rate of forgetting

A repeated measures ANOVA, entering the short delay free recall and long delay free recall scores from CVLT as within-subject factors, showed no statistically significant difference between the two APOE groups.

An analysis of the rate of forgetting, including the learning trial 5 and the delayed free recall score from CVLT as between subject factors, showed no statistically significant APOE group difference.

### Recognition

Mann-Whitney nonparametric tests were used to analyze the differences between ε4 carriers and non-carriers on recognition measures. The only significant difference appeared for the false alarm scores (MW, *p *= 0.02, *r *= -0.27).

### Effects of gender, age, and dose on cognitive performance

To examine modulating effects of gender and age on CVLT performance, we performed repeated measures ANOVAs including APOE status and gender as between subject factors and learning trial 1 to trial 5 as within subject factors. Even though several mean values indicated that women performed better than men, the analysis revealed no statistically significant interaction effect of APOE status and gender (F (1,68), *p *= 0.09). When rerunning the analysis by including age and APOE status as between subject factors, no statistically significant effect (F (1,68), *p *= 0.52) was demonstrated. The analyses of dose effects showed no statistically significant differences between homozygotes and heterozygotes for ε4 on any measure of learning and memory function.

### aMCI

There were 32 participants who were allocated to the group of aMCI according to our definition (i.e. a free recall performance at least 1.5 standard deviations below normative mean), 6 homozygotes, 18 heterozygotes and 8 non-carriers of the ε4 allele. Test statistics revealed a statistically significant higher number of ε4 carriers than non-carriers in this aMCI group (χ^2 ^= 4.06, *p *= 0.04). The average age in the aMCI group was slightly higher (M = 65.1, SD = 8.1) than the non-aMCI group (M = 62.8, SD = 7.3), but the difference was not statistically significant (*t *= -0.236, *p *= 0.221). However, individuals in the aMCI group performed significantly lower on the MMSE (aMCI M = 27.3 (SD = 1.7), non-aMCI M = 28.4 (SD = 1.7), *t *= 2.58, *p *= 0.01) and had a lower IQ score (aMCI M = 101 (SD = 11.9), non-aMCI M = 110 (SD = 15.1), *t *= 2.61, *p *= 0.01). There was no statistically significant group difference with respect to educational level (aMCI M = 10.4 (SD = 2.7), non-aMCI M = 11.8 (SD = 3.2), *t *= 1.85, *p *= 0.07).

## Discussion

Results from the present study showed that the APOE ε4 carriers showed a statistically significant lower performance on a number of measures of verbal learning and memory than the non-carriers. Performance of the ε4 carriers on the CVLT was characterized by fewer correctly reported words on all learning trials, reduced between-trial recall consistency, a higher number of intrusions during learning trials, and an increased frequency of false alarms in recognition trials than the non-carriers. Recall of the verbal material across varying delays was not disproportionately reduced in the ε4 carriers, but further statistically significant differences between the two groups were demonstrated for the Boston Naming Test, the recall measure of the RCFT, and on the MMSE. Gross measures of cerebral atrophy or subtle vascular pathology on MRI could not distinguish between ε4 carriers and non-carriers, and the level of subjective complaint of memory problems was not significantly related to APOE status. According to the selected criteria for MCI, 46% of the participants were classified as aMCI. The aMCI group showed an increased frequency of ε4 alleles than the non-MCI group.

The results were generally in accordance to those in previous studies of clinically recruited aging participants, showing that APOE ε4 is associated with impaired performance on subtasks from the CVLT [13,14]. There were, however, differences in the profile of test results between the study groups. Bondi et al. [14] found that reduced delayed recall and learning slope discriminated well between the APOE groups, whereas our results indicated that a pattern of inconsistent retrieval and erratic reporting (intrusions of non-presented words) characterized the learning and memory function in ε4 carriers. In spite of the fact that the CVLT measure of long delay recall has shown to be sensitive to early changes of AD in other studies [13,14,48], the two APOE groups showed similar results in the our study. This might be due to the lack of statistical power because of the small sample size in the present study. This argumentation was supported by medium effect sizes on scores such as long delay free recall (CVLT) and the Verbal Paired Association Test (learning and recall). Furthermore, the age range in the present sample may have influenced our results. The participants were on average 7 years younger than the ones studied by Bondi et al. [14] (64 years vs. 71 years). We suggest that the younger age of the participants in the present study may partly explain why their memory performances were less characteristic of mild AD than the patient group studied by Bondi et al. [14]. Even if several studies concur that measures of delayed recall are predictive of risk for developing dementia [14,48], other have found that measures of learning are equally or more predictive [49,50]. However, the distinction between learning and recall may not be very informative since episodic memory is critically involved both in learning performances across repeated trials and in delayed recall.

In the present study, the APOE status was found to have a significant effect on the recall measure from the RCFT. This was not found in the Bondi et al. [14] study, although the performance on another visual memory test (Wechsler Memory Scales, visual reproduction) showed a trend towards lower scores in ε4 carriers. Other studies of ε4 carriers which have included the RCFT have shown mixed results [51,52], and our finding was in accordance with the study of Caselli et al. [18], showing lower delayed recall sores for ε4 carriers on a complex figure test.

An atypical finding in our study was the poorer performance of the ε4 carriers on the first presentation of the word list. This may be explained as an attention deficit, a word finding or semantic deficit, or a memory problem. Performance on the Boston Naming Test differed significantly between ε4 carriers and non-carriers, which could suggest a word retrieval problem. However, we believe that this was not the case since both groups obtained results that were more than one standard deviation above age-corrected norms (not shown data) and no other measures of verbal function (Vocabulary, animal and letter fluency) confirmed such a problem in the ε4 carriers. Together with the lack of group differences on the remaining tests of attention (Digit Symbol Substitution, Trail Making Test, Stroop Color Word), we suggest a selective memory problem in the ε4 carriers.

The significant lower performance of ε4 carriers than the non-carriers on the MMSE deserves a comment, because such a finding could indicate that ε4 carriers showed general lower cognitive functioning than the non-carriers. We argue against such a conclusion, in that the two groups performed almost equal on the comprehensive neuropsychological test battery, expected to be at least as sensitive to impairment as the MMSE. Furthermore, the MMSE difference was less than one point and all participants obtained a MMSE score above 24.

It may be argued that the displayed problems in the ε4 carriers are a consequence of the inclusion of patients with subjective memory problems. This argument may have some merit if the study population is defined on the basis of reduced performance on memory tests, as is the case in some definitions of MCI [3]. In the present study, we included participants on the basis of widely defined clinical criteria based on subjective cognitive complaints, with symptoms and pathological markers of dementia as exclusion criteria. In view of the high incidence of subjective memory complaints in the general population [5], it is likely that patients recruited on the basis of this criterion will include a heterogeneous group of normal and mildly pathological cases. Our results confirmed that subjective memory complaint is not associated with APOE status [53], and Fisk et al. [6] found that eliminating the criterion of subjective memory complaint from the definition of MCI had no impact on the relative risk of subsequent cognitive decline. Therefore, it is less likely that subjective memory complaint has served as a strong factor biasing the findings of an association of memory dysfunction and APOE ε4 in the present study.

Despite the relatively young age of our sample, 46% of the patients satisfied psychometric criteria for aMCI. This group showed an increased frequency of APOE ε4 alleles, and data from other studies [3,54] suggest that the aMCI group has an increased risk of developing AD. Thus, the association between APOE status and memory performance may be explained by the presence of a significant number of preclinical AD patients in the study population. This was also the conclusion drawn by Bondi et al. [13,14] on the basis of follow-up data for their sample. We acknowledge that this is a likely explanation for part of our findings. Although these authors showed that the combination of impaired memory performance and APOE ε4 genotype had a high predictive value in the course to AD, this has not always been confirmed [49,55]. It is important to note that in the present study, 25% of the participants classified as aMCI were non-carriers. This possibly reduces their risk to progress to AD. Furthermore, this points to an important weakness of the concept of MCI. A number of authors have demonstrated that elderly individuals showed fluctuating performance when assessed with neuropsychological tests [56], and that there are individuals classified as MCI who remained stable or even re-obtained normal cognitive function on follow-up [57]. In our group, other reasons than underlying neurodegenerative processes could have lead to classification as aMCI. In the present study, the group of individuals defined as aMCI had significant lower IQ scores and lower scores on the MMSE. Thus, some of individuals in the aMCI group may have been misclassified due to a general low cognitive abilities or a more a temporary cognitive impairment.

Proximity to a likely age of debut for AD is expected to be a powerful factor predicting memory deficit. Although the average age for the aMCI group was somewhat higher than the remaining group, we did not find that age as such was a significant modulator of the relation between APOE status and memory performance. Homozygozity for ε4 has been shown to confer another significant increase in risk for conversion to AD, but this was not confirmed by the results in the present study. In this respect our results resemble those of Caselli et al. [17] in a normal group in an age range similar to ours. In accordance with Caselli's group [18,58] we noted that while the definition of aMCI emphasizes a memory profile resembling mild AD with reduction in delayed recall, this was not the pattern of memory performance that best characterized the difference between ε4 carriers and non-carriers in the present group. Based on all these observations we assume that the whole study group rather than a subgroup with early AD has contributed to the results in the present study. While the APOE ε4 related mechanisms that are not explicitly linked to AD pathology [15] are thought to affect neuronal health in general, it is likely that memory and learning are domains of cognitive function that are more sensitive to impaired synaptic plasticity than other domains. More sensitive and specific tests of attention, as shown by Greenwood and Parasuraman using a cued visual discrimination task [23], may also reveal effects in this domain.

Even though we used Bonferoni correction for a number of analyses, this study can be criticized for the use of multiple comparisons since some of the neuropsychological measures surely tap the same cognitive resources. This and the small sample size request caution when interpreting the results until these are verified by further North European studies. Another issue is the clinical impact of our findings. In the present study, the aim was to investigate group differences rather than individual patterns of cognitive function. Although group results give indications to a clinician, he or she will still have to assess their patients individually to take into account the individual differences in older adults. Follow-up studies are necessary to answer the question of the predictive value of cognitive decline in patients with APOE ε4 in Nordic samples and characteristics of different developmental pathways.

## Conclusion

The present study of consecutively referred non-demented patients included 55% of participants with at least one ε4 allele, and 15 % of the total sample were ε4 homozygotes. The proportion is high in comparison with MCI samples [4] and with unselected AD cases [59], which reflect the fact that the general population from which the present patients were recruited also has a high incidence of ε4 alleles [26]. While APOE has been commonly accepted as a susceptibility gene for late onset AD, with increased risk associated with the ε4 allele, it has also become increasingly clear that genetic risk is modulated by other factors. There is increasing information of gene combinations that may serve to modify APOE effects [24,60]. While the present findings are largely consistent with reports based on North American and European populations, further comparative studies of effects on cognition of APOE and other genes may give information relevant to focusing on APOE. mechanisms as a therapeutic target. Exploiting the natural variation in prevalence of at-risk alleles is an important part of this strategy.

## Competing interests

The author(s) declare that they have no competing interests.

## Authors' contributions

EW conducted data analyses, literature review and prepared drafts of the manuscript; ALJ instigated data analyses and contributed to the interpretation of results and drafts of the manuscript; BS and LG examined all participants and made diagnostic judgments; IR was responsible for the design and data collection, data analyses, literature review, and contributed substantially to the manuscript. All authors have read and approved the final manuscript and contributed equally to this work

## References

[B1] Lobo A, Launer LJ, Fratiglioni L, Andersen K, Di Carlo A, Breteler MM, Copeland JR, Dartigues JF, Jagger C, Martinez-Lage J, Soininen H, Hofman A (2000). Prevalence of dementia and major subtypes in Europe: A collaborative study of population-based cohorts. Neurologic diseases in the elderly research group. Neurology.

[B2] Morris JC, Storandt M, Miller JP, McKeel DW, Price JL, Rubin EH, Berg L (2001). Mild cognitive impairment represents early-stage Alzheimer disease. Arch Neurol.

[B3] Petersen RC (2004). Mild cognitive impairment as a diagnostic entity. J Intern Med.

[B4] Arnaiz E, Almkvist O, Ivnik RJ, Tangalos EG, Wahlun d LO, Winblad B, Petersen RC (2004). Mild cognitive impairment: a cross-national comparison. J Neurol Neurosurg Psychiatry.

[B5] Koivisto K, Reinikainen KJ, Hanninen T, Vanhanen M, Helkala EL, Mykkanen L, Laakso M, Pyorala K, Riekkinen PJ (1995). Prevalence of age-associated memory impairment in a randomly selected population from eastern Finland. Neurology.

[B6] Fisk JD, Merry HR, Rockwood K (2003). Variations in case definition affect prevalence but not outcome in mild cognitive impairment. Neurology.

[B7] Tervo S, Kivipelto M, Hanninen T, Vanhanen M, Hallikainen M, Mannermaa A, Soininen H (2004). Incidence and risk factors for mild cognitive impairment: a population-based three-year follow-up study of cognitively healthy elderly subjects. Dement Geriatr Cog Disorders.

[B8] Raber J, Huang Y, Wesson Ashford J (2004). APOE accounts for the vast majority of AD risk and AD pathology. Neurobiol Aging.

[B9] Blacker D, Haines JL, Rodes L, Terwedow H, Go RC, Harrell LE, Perry RT, Barret SS, Chase G, Meyers D, Albert MS, Tanzi R (1997). APOE 4 and age at onset of Alzheimer's disease: the NIMH genetics initiative. Neurology.

[B10] Farrer LA, Cupples LA, Haines JL, Hyman B, Kukull WA, Mayeux R, Myers RH, Pericak-Vance MA, Risch N, vanDuijn CM (1997). Effects of age, sex, and ethnicity on the association between apolipoprotein E genotype and Alzheimer disease. A meta-analysis. APOE and Alzheimer Disease Meta Analysis Consortium. JAMA.

[B11] Farlow MR, He Y, Tekin S, Xu J, Lane R, Charles HC (2004). Impact of APOE in mild cognitive impairment. Neurology.

[B12] Smith GE, Bohac DL, Waring SC, Kokmen E, Tangalos EG, Ivnik RJ, Petersen RC (1998). Apolipoprotein E genotype influences cognitive 'phenotype' in patients with Alzheimer's disease but not in healthy control subjects. Neurology.

[B13] Bondi MW, Salmon DP, Monsch AU, Galasko D, Butters N, Klauber MR, Thal LJ, Saitoh T (1995). Episodic memory changes are associated with the APOE-epsilon 4 allele in nondemented older adults. Neurology.

[B14] Bondi MW, Salmon DP, Galasko D, Thomas RG, Thal LJ (1999). Neuropsychological function and apolipoprotein E genotype in the preclinical detection of Alzheimer's disease. Psychol Aging.

[B15] Mahley RW, Weisgraber KH, Huang Y (2006). Apolipiprotein E4: A causative factor and therapeutic target in neuropathology, including Alzheimer's disease. Proc Natl Acad Sci USA.

[B16] Small BJ, Rosnick CB, Fratiglioni L, Backman L (2004). Apolipoprotein E and cognitive performance: a meta-analysis. Psychol Aging.

[B17] Mayeux R, Small SA, Tang M, Tycko B, Stern Y (2001). Memory performance in healthy elderly without Alzheimer's disease: effects of time and apolipoprotein-E. Neurobiol Aging.

[B18] Caselli RJ, Reiman EM, Osborne D, Hentz JG, Baxter LC, Hernandez JL, Alexander GG (2004). Longitudinal changes in cognition and behavior in asymptomatic carriers of the APOE ε4 allele. Neurology.

[B19] Mortensen EL, Hogh PA (2001). Gender difference in the association between APOE genotype and age-related cognitive decline. Neurology.

[B20] Reiman EM, Caselli RJ, Yun LS, Chen K, Bandy D, Minoshima S, Thibodeau SN, Osborne D (1996). Preclinical evidence of Alzheimer's disease in persons homozygous for the epsilon 4 allele for apolipoprotein E. New Engl J Med.

[B21] Bookheimer SY, Strojwas MH, Cohen MS, Saunders AM, Pericak-Vance MA, Maziotta JC, Small GW (2000). Pattern of brain activation in people at risk for Alzheimer's disease. New Engl J Med.

[B22] Lind J, Persson J, Ingvar M, Larsson A, Cruts M, Van Broeckhoven C, Adolfsson R, Backman L, Nilsson LG, Petersson KM, Nyberg L (2006). Reduced functiobnal brain activity response in cognitively intact apolipoprotein E ε4 carriers. Brain.

[B23] Greenwood PM, Parasuraman R (2003). Normal genetic variation, cognition, and aging. Behav Cogn Neurosci Rev.

[B24] Espeseth T, Greenwood P, Reinvang I, Fjell AM, Walhovd KB, Westlye LT, Wehling E, Lundervold AJ, Rootwelt H, Parasuraman R (2006). Interactive effects of APOE and CHRNA4 on attention and white matter volume in healthy middle aged and older adults. Cogn Affect Behav Neurosci.

[B25] Gureje O, Ogunniyi A, Baiyewu O, Price B, Unverzagt F, Evans RM, Smith-Gamble V, Lane KA, Gao S, Hall KS, Hendrie HC, Murrell JR (2006). APOE ε4 is not associated with Alzheimer's Disease in elderly Nigerians. Ann Neurol.

[B26] Gerdes LU (2003). The common polymorphism of apolipoprotein E: geographical aspects and new pathophysiological relations. Clin Chem Lab Med.

[B27] Berr C, Wancata J, Ritchie K (2005). Prevalence of dementia in the elderly in Europe. Eur Neuropsychopharmacol.

[B28] Fratiglioni L, Launer LJ, Andersen K, Breteler, MED MER, Copeland JR, Dartigues J-F, Lobo A, Martinez-Lage J, Soininen H, Hofman A (2000). Incidence of dementia and major subtypes in Europe: A collaborative study. Neurology.

[B29] Welsh KA, Butters N, Mohs RC, Beekly D, Edland S, Fillenbaum G, Heyman A (1994). The Consortium to Establish a Registry for Alzheimer's Disease (CERAD). Part V. A normative study of the neuropsychological battery. Neurology.

[B30] Sundet K, Lundervold AJ (2004). CVLT-Norsk versjon. Sollentuna, Psykologiförlaget AB.

[B31] Folstein MF, Folstein SE, McHugh PR (1995). "Mini-mental state". A practical method for grading the cognitive state of patients for the clinician. Journal Psychiatr Res.

[B32] American Psychiatric Association (1994). Diagnostic and statistical manual of mental disorders. DSM-IV.

[B33] World Health Organization (1993). The ICD-10 Classification of Mental and Behavioral Disorders.

[B34] World Medical Association (1993). Declaration of Helsinki. Recommendations guiding physicians in biomedical research involvinghuman subjects. J Med Assoc.

[B35] Lezak M, Howieson DB, Loring DW (1993). Neuropsychological assessment.

[B36] Wechsler D (1999). Wechsler Abbreviated Scales of Intelligence.

[B37] Delis D, Kramer J, Kaplan E, Ober B (1987). The California Verbal Learning Test.

[B38] Andersen R (1976). Verbal and visuo-spatial memory. Two clinical tests administered to a group of normal subjects. Scand J Psychol.

[B39] Osterrieth PA (1944). Le test de copie d'une figure complexe. Archives de Psychologie.

[B40] Wechsler D (1981). Wechsler Adult Intelligence Scale – Revised manual.

[B41] Reitan RM, Davidson LA (1974). Clinical Neuropsychology: Current Status and Applications.

[B42] Jensen AR, Rohwer WD (1966). The Stroop Color-Word Test: A review. Acta Psychologica.

[B43] Benton AL, Hamsher K (1989). Multilingual Aphasia Examination.

[B44] Kaplan EF, Goodglass H, Weintraub S (1989). The Boston Naming Test.

[B45] Frisoni GB, Scheltens P, Galluzzi S, Nobili FM, Fox NC, Robert PH, Soininen H, Wahlund LO, Waldemar G, Salmon E (2003). Neuroimaging tools to rate regional atrophy, subcortical cerebrovascular disease, and regional cerebral blood flow and metabolism: consensus paper of the EADC. J Neurol Neurosurg Psychiatry.

[B46] SPSS (2006). SPSS for Windows. [Release 14.0].

[B47] Cohen J (1992). A power primer. Psychol Bull.

[B48] Tierney MC, Yao C, Kiss A, McDowell I (2005). Neuropsychological tests accurately predict incident Alzheimer disease after 5 and 10 years. Neurology.

[B49] Albert MS, Moss MB, Tanzi R, Jones K (2001). Preclinical prediction of AD using neuropsychological tests. J Int Neuropsychol Soc.

[B50] Grober E, Kawas C (1997). Learning and retention in preclinical and early Alzheimer's disease. Psychol Aging.

[B51] Chey J, Kim JW, Cho HY (2000). Effects of apolipoprotein E phenotypes on the neuropsychological functions of community-dwelling elderly individual without dementia. Neurosci Lett.

[B52] Cohen RM, Small C, Lalonde F, Friz J, Sunderland T (2001). Effect of apolipoprotein E genotype on hippocampal volume loss in aging healthy women. Neurology.

[B53] Lautenschlager NT, Flicker S, Vasikaran S, Leedman P, Almeida OP (2005). Subjective memory complaintes with and without objective memory impairment: Relationship with risk factors for dementia. Am J Geriatr Psychiatry.

[B54] Petersen RC, Smith GE, Waring SC, Ivnik RJ, Tangalos EG, Kokmen E (1999). Mild cognitive impairment: clinical characterization and outcome. Arch Neurol.

[B55] Devanand DP, Pelton GH, Zamora D, Liu X, Tabert MH, Goodkind M, Scarmeas N, Braun I, Stern Y, Mayeux R (2005). Predictive utility of Apolipoprotein E genotype for Alzheimer Disease in outpatients with mild cognitive impairment. Arch Neurol.

[B56] Palmer BW, Boone KB, Lesser IM, Wohl MA (1998). Base rates of impaired neuropsychological test performance among healthy older adults. Arch Clin Neuropsychol.

[B57] deRetrou J, Wenisch E, Chausson C, Dray F, Faucounau V, Rigaud AS (2005). Accidental MCI in healthy subjects: a prospective longitudinal study. Eur J Neurol.

[B58] Caselli RJ, Osborne D, Reiman EM, Hentz JJG, Barbieri CJ, Saunders AM, Hardy J, Graff-Radford NR, Hall GR, Alexander GE (2001). Preclinical cognitive decline in late middle-aged asymptomatic Apoliprotein E-ε4/4 homozygotes: A replication study. J Neurol Sci.

[B59] Rubinsztein DC, Easton DF (1999). Apolipoprotein E genetic variation and Alzheimer's disease: a meta-analysis. Dem Ger Cogn Dis.

[B60] Borroni B, Archetti S, Agosti C, Akkawi N, Brambilla C, Caimi L, Caltagirone C, DiLuca M, Padovani A (2005). Intronic CYP46 polymorphism along with APOE genotype in sporadic Alzheimer Disease: from risk factors to disease modulators. Neurobiol Aging.

